# Grain Structure Engineering of NiTi Shape Memory Alloys by Intensive Plastic Deformation

**DOI:** 10.1021/acsami.2c05939

**Published:** 2022-06-27

**Authors:** Zifan Wang, Jingwei Chen, Radim Kocich, Samuel Tardif, Igor P. Dolbnya, Lenka Kunčická, Jean-Sébastien Micha, Konstantinos Liogas, Oxana V. Magdysyuk, Ivo Szurman, Alexander M. Korsunsky

**Affiliations:** † MBLEM, Department of Engineering Science, University of Oxford, Oxford OX1 3PJ, U.K.; ‡ Faculty of Mechanical Engineering, Brno University of Technology, Technická 2896/2, Brno 61669, Czech Republic; § Université Grenoble Alpes, CEA-Grenoble/IRIG, 38043 Grenoble Cedex 9, France; ∥ CRG-IF BM32 Beamline, European Synchrotron Radiation Facility, 38043 Grenoble Cedex 9, France; ⊥ Diamond Light Source, Harwell Campus, Oxfordshire OX11 0DE, U.K.; # Faculty of Materials Science and Technology, VŠB-Technical University of Ostrava, Ostrava 708 00, Czech Republic

**Keywords:** bespoke NiTi shape memory alloys, grain structure, multiscale, lattice rotation, phase transformation, Laue microdiffraction, powder diffraction

## Abstract

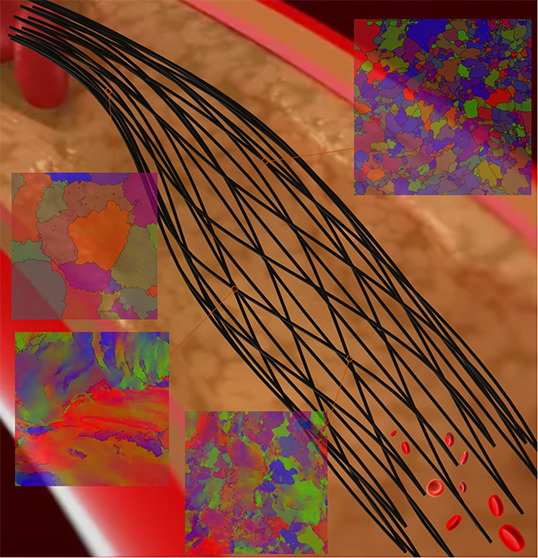

To explore an effective route of customizing the superelasticity (SE) of NiTi shape memory alloys via modifying the grain structure, binary Ni_55_Ti_45_ (wt) alloys were fabricated in as-cast, hot swaged, and hot-rolled conditions, presenting contrasting grain sizes and grain boundary types. *In situ* synchrotron X-ray Laue microdiffraction and *in situ* synchrotron X-ray powder diffraction techniques were employed to unravel the underlying grain structure mechanisms that cause the diversity of SE performance among the three materials. The evolution of lattice rotation, strain field, and phase transformation has been revealed at the micro- and mesoscale, and the effect of grain structure on SE performance has been quantified. It was found that (i) the Ni_4_Ti_3_ and NiTi_2_ precipitates are similar among the three materials in terms of morphology, size, and orientation distribution; (ii) phase transformation happens preferentially near high-angle grain boundary (HAGB) yet randomly in low-angle grain boundary (LAGB) structures; (iii) the smaller the grain size, the higher the phase transformation nucleation kinetics, and the lower the propagation kinetics; (iv) stress concentration happens near HAGBs, while no obvious stress concentration can be observed in the LAGB grain structure during loading; (v) the statistical distribution of strain in the three materials becomes asymmetric during loading; (vi) three grain lattice rotation modes are identified and termed for the first time, namely, multi-extension rotation, rigid rotation, and nondispersive rotation; and (vii) the texture evolution of B2 austenite and B19′ martensite is not strongly dependent on the grain structure.

## 1 Introduction and Objectives

Shape memory alloys (SMAs) are probably the most important category of shape memory materials. In recent years, the utilization of SMAs has been extended to cutting-edge engineering applications, including, but not limited to, medical devices, actuators, micromechanical systems, deployable structures, and elastocaloric devices.^1^ Among the three prevalent SMA systems, viz. NiTi-based, Cu-based, and Fe-based, the binary NiTi alloy is often preferred in engineering applications because of its excellent functional performance and biocompatibility.^2^

Two extensively utilized aspects of the functional behavior of NiTi alloys are the shape memory effect (SME) and superelasticity (SE). SME is commonly used in actuators and deployable structures, while SE has wider application fields, the most popular ones being medical stents and elastocaloric cooling systems. The latter SE effect falls within the focus of the present study. To induce SE, the material operation temperature should be kept above the critical temperature at which the major phase is austenite. When stress is applied, cubic austenite transforms to monoclinic martensite in a nondiffusive way, thus pseudo-plastically accommodating the deformation. After stress is withdrawn, reverse phase transformation occurs so that the component recovers to its original shape.

A critical challenge concerning NiTi alloys awaiting to be resolved is to find a reliable method to customize its SE performance for different application scenarios. For instance, the phase transformation stress needs to be as low as possible in elastocaloric cooling devices and to be at a moderate level in medical stents. When used in orthodontics, a gradually increased phase transformation stress during deformation is required so that the component acts like a spring, while in a medical stent, a stress plateau would be favored to apply constant pressure to the vascular wall. In reality, numerous studies have found that this stress-induced phase transformation process can be affected by many microstructural factors: grain size,^3^ texture,^1,4^ composition ratio,^5^ dislocation density,^1,6^ tertiary element,^7^ and precipitation.^8^ Furthermore, the thermomechanical processing history can also be influential, namely, the fabrication route^7,9^ and heat treatment.^8,10^

The aforementioned studies and others not mentioned here appear to fail to explore the effect of grain structure and the possibility of customizing the SE through grain structure engineering. Besides that, most studies were based on *ex situ* rather than *in situ* experiments, so that the underlying mechanisms of how these factors influence SE performance were somewhat superficially investigated. The term “microstructure” refers to the rain morphology and includes the overall properties of grains: the grain boundary type, morphology, and size. Grain boundary type can be classified into two categories, viz. high-angle grain boundary (HAGB) and low-angle grain boundary (LAGB).

*In situ* synchrotron X-ray Laue microdiffraction (μLaue) may be the most advantageous technique in probing the microscale evolution of microstructures at the scale up to a few thousands of microns with a typical resolution of one micron. White X-ray beam is focused to a spot size of less than one micron, illuminating a volume depth of approximately ten microns in individual crystallites; thus, the diffraction pattern contains valuable crystallographic information, namely, crystal orientation, microdefects, and local elastic strain. Due to the simplicity of the setup, great flexibility can be offered for the use of sample environment equipment, such as heating stages and loading rigs. It has to be pointed out that although μLaue has always been compared with HR-EBSD, which can provide similar crystallographic information, the former is undoubtedly superior:^11^ the elastic strain resolution of μLaue is 10^–5^, while that of HR-EBSD is 10^–4^; the orientation resolution of μLaue is <0.01°, while that of HR-EBSD is ∼0.01°; the sample environment of μLaue can be of any type, while HR-EBSD must be in the vacuum; and most importantly, the penetration depth of μLaue is tens of microns, while that of HR-EBSD is merely a few nanometers. Therefore, μLaue provides much more representative information of the probed bulky crystal.

*In situ* synchrotron high energy X-ray powder diffraction (HE-XRD) has been universally acknowledged to be one of the most advanced techniques in probing the evolution of microstructures. The diffraction pattern contains crystallographic information including texture, phase fraction, elastic strain, microdefects, and lattice *d*-spacing. The superiority of this technique has been elucidated in numerous studies; for details, please refer to ref (12).

Having identified the significance of customizing SE performance in NiTi SMA and the boundary of existing studies, the objective of this work is to tailor the SE effect in NiTi SMA via grain structure engineering, namely, via the modification of grain boundary type and grain size. NiTi samples of different grain sizes were fabricated by two processing routes: as-cast (AC) and hot swaging (HS). The grain boundary type of these two samples is dominated by HAGB. The other NiTi sample was manufactured by hot rolling (HR), in which the majority of grain boundaries are LAGBs, and very few HAGBs can be observed. These three NiTi SMAs exhibit diverse SE performances. *In situ* synchrotron X-ray Laue microdiffraction and *in situ* synchrotron X-ray powder diffraction techniques were employed to unravel the underlying grain structure effects on the deformation mechanisms. The aspects of multiscale lattice rotation, strain field, and phase transformation behavior of different grain structures were quantified, and the effect of grain structure on SE performance was comprehensively evaluated. Other supplementary probing and simulation techniques were also employed to support the findings, including DSC, FIB-SEM, TEM, TEM-EDX, EBSD, and CPFE.

The significance of the present study lies in it being probably the first theoretical and practical guidance that opens up new possibilities to customize the superelasticity of NiTi shape memory alloys by means of grain structure engineering, thereby vastly broadening the application scenarios in deployable structures which require precise control of the reaction force associated with the superelasticity effect.

## 2 Experimental and Analytical Details

### 2.1 Material Processing Methodology

Nickel cubes of 99.99% purity and Grade 2 titanium of >99.3% purity were used as raw materials. A polycrystalline Ni_55_Ti_45_ wt % ingot was cast in an isostatically pressed graphite crucible via vacuum induction melting (VIM) under 99.9999% purity argon protection at 150 Mbar.

The as-cast ingot was split into three pieces. The first one remained in the as-cast (AC) state. The second piece was subjected to hot rotary swaging (HS) at 950 °C using the self-developed KOMAFU S600 system;^13,14^ for details of this manufacturing technique, please refer to.^1,15,16^ The last piece was subjected to hot rolling (HR) at 950 °C; for details, please refer to.^2,9^ Manufacturing details for grain structure engineering are elucidated in Appendix A.

All three pieces were cut into flat dog-bone-shaped samples using electrical discharge machining (EDM) and heat-treated at 900 °C for 4 h in a vacuum furnace to minimize the amount of lattice defects and to produce well-crystallized microstructures. Immediately after heat treatment, all samples were water-quenched to room temperature. Two samples from each sample state, namely, AC, HS, and HR, were used in two different *in situ* experiments. Since the two samples were originally one piece and separated by EDM, they are considered as possessing identical initial material properties.

### 2.2 Differential Scanning Calorimetry Analysis

**Figure 1:**
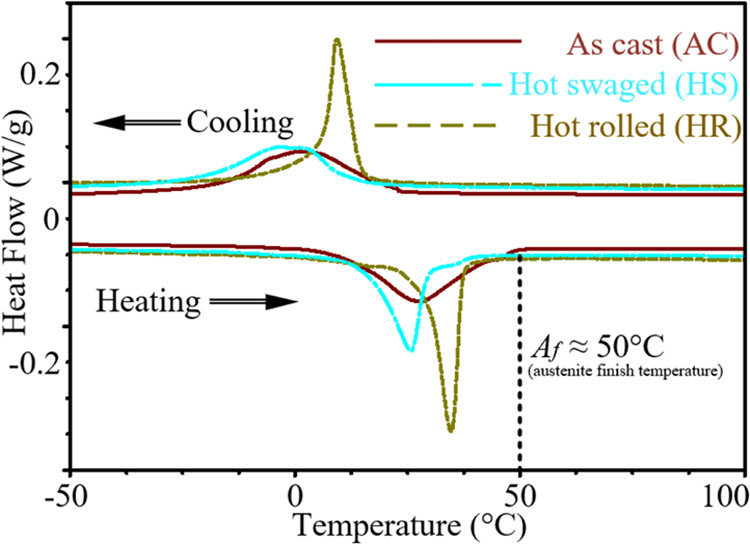
DSC measurement of critical temperatures for thermally induced phase transformation in AC, HS, and HR NiTi.

To ensure the samples contained a maximal fraction of B2 austenite phase prior to superelastic deformation, austenite finish temperatures of the three sample states were measured by a TA DSC Q2000 instrument. The measurements were performed between −90 and 200 °C at a ramp rate of ±5 °C/min. The results are shown in Figure 1. All materials possess the austenite finish temperature (*A*_f_) at approx. 50 °C, above which the materials contain the maximal fraction of the B2 austenite phase. For detailed explanations of *A*_f_ as well as the other critical phase transformation temperatures of NiTi alloys, please refer to.^8^ Judging from that, for all three NiTi materials, the *in situ* superelastic loading experiments were conducted at 50 °C.

### 2.3 Microscopy Characterization

To investigate the microstructural effect of the material processing method and to identify the suitable mapping area for *in situ* Laue microdiffraction, electron backscatter diffraction (EBSD) observations were conducted in a Tescan LYRA3 FIB/SEM system integrated with a SYMMETRY EBSD detector from Oxford Instruments. Samples were finely polished by gradually decreasing the size of the diamond particle suspension from 9 to 0.25 μm colloidal silica. Kikuchi pattern indexing was carried out at an accelerating voltage of 20 kV, a working distance of 9 mm, a step size of 0.5 μm, and an acquisition speed of 5 Hz.

Transmission electron microscopy (TEM) lamellae of thickness ∼120 nm were prepared by a refined lift-out technique^17^ in the above-mentioned LYRA3 system. Observations were carried out at ePSIC at the Diamond Light Source, using a JEOL ARM200CF system at 200 kV. Composition ratios of precipitates and the matrix were quantified by the TEM-EDX technique by focusing at the precipitation region and matrix region in the elemental map.

### 2.4 *In situ* Synchrotron X-ray Laue Microdiffraction (μLaue)

**Figure 2:**
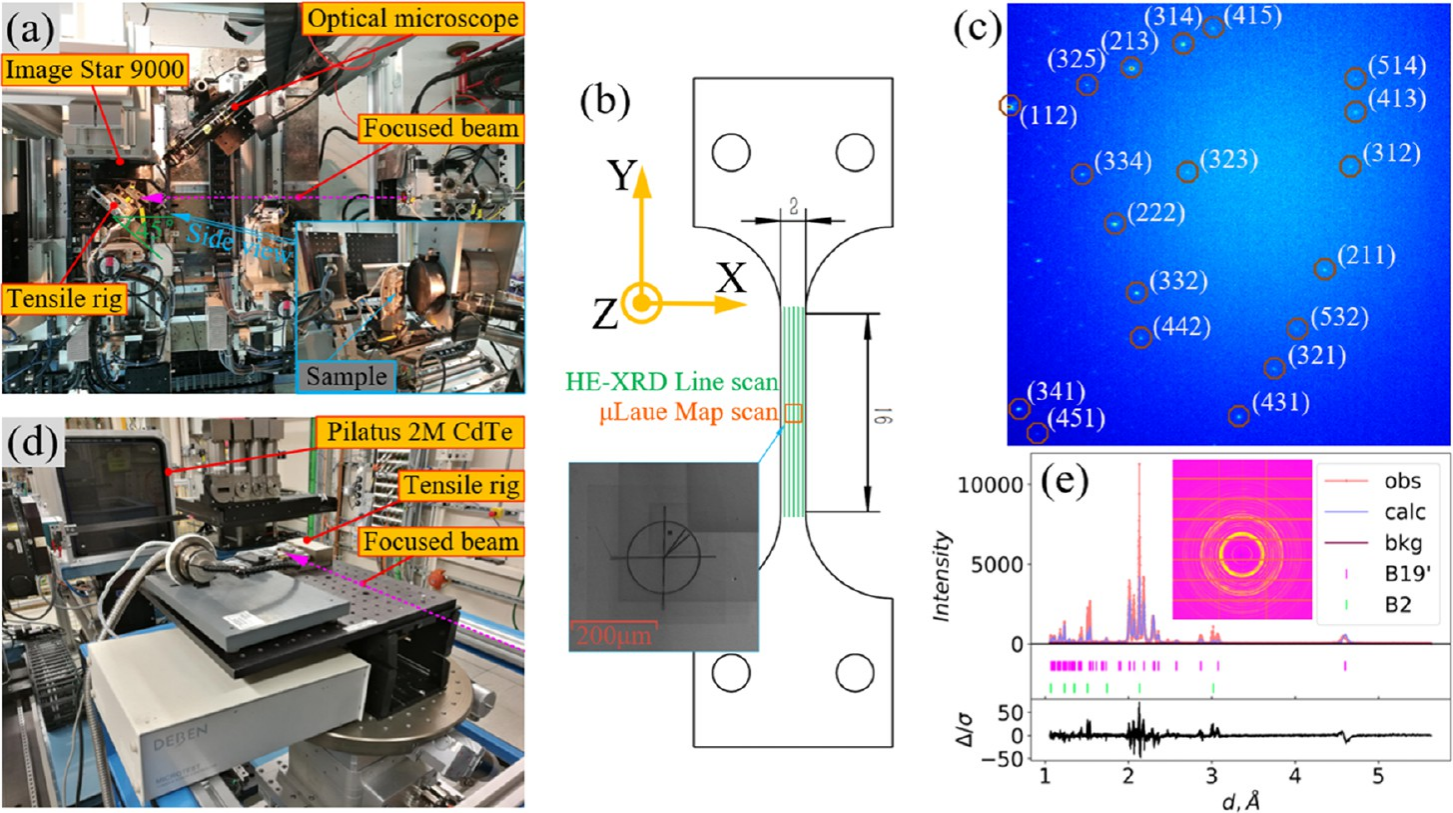
(a) Experimental setup for *in situ* synchrotron X-ray Laue microdiffraction (μLaue). (b) Sample geometry (unit: mm). Laue microdiffraction map scan is indicated by a FIB marker at the corner, overlapping with the EBSD map. HE-XRD line scan covers the whole gauge length of the sample. (c) Typical indexed Laue microdiffraction pattern for the NiTi B2 austenite phase. (d) Experimental setup for *in situ* synchrotron high-energy X-ray powder diffraction (HE-XRD). (e) Example of integrated HE-XRD pattern; both B2 austenite and B19′ martensite were identified and refined via the Rietveld method.

*In situ* μLaue experiments were performed at two synchrotron facilities. At the CRG-IF BM32 beamline at ESRF,^18^ a polychromatic X-ray beam with an energy range of 5–25 keV was focused by a set of Kirkpatrick–Baez (KB) mirrors to a typical size <0.8 μm. At the B16 test beamline at DLS,^19^ the white beam with an energy range of 8–30 keV was collimated to a size of ∼5 to 10 μm, using a nearly identical experimental setup as illustrated in Figure 2a.

The flat dog-bone-shaped samples were clamped on a 5 kN DEBEN tensile rig integrated with a heating stage to maintain the sample temperature at 50 °C. An extensometer was also attached to the sample. The rig was mounted in such a way that the sample surface was inclined 45° relative to the incident focused beam. The center position of the 2D X-ray large-area CCD detector (ImageStar 9000, Photonic Science Ltd.) was aligned at a 2θ position of ∼90°, and the distance between the detector and the sample was brought as close as 60 mm to capture the maximal number of Laue diffraction spots. The optical microscope was fixed, whose line of sight coincided with the incident beam. A single-crystal germanium of orientation 111 was used for precise detector geometry calibration.

Prior to the experiments, the designated surface region was selected from corresponding EBSD maps and marked with a “cross-circle” sign at the square region corner as shown in Figure 2b. The sign was readily observable under the optical microscope and therefore was used as a reference to assure the same *in situ* μLaue map scan area during incremental tensile loading levels.

Since the beam size is significantly smaller than the grain size observed from EBSD, only one grain was illuminated in the vast majority of the scanning points. A representative μLaue diffraction pattern for the body-centered cubic NiTi B2 austenite phase is shown in Figure 2c, indexed using LaueTools software.^20^ Upon further refinement of the indexed diffraction spots, all six components of the deviatoric elastic strain tensor and three Euler angles of crystallographic orientation can be determined with superior accuracy.^18^ As an initial input to LaueTools, the lattice parameters for the cubic B2 phase of the undeformed virgin state were determined by Rietveld refinement of the time-of-flight neutron diffraction spectrum in our previous studies.^1,21^ The visualization of μLaue maps was achieved by the MTEX package.^22^

### 2.5 *In Situ* Synchrotron High-Energy X-ray Powder Diffraction (HE-XRD)

*In situ* HE-XRD measurements were carried out at the I12 JEEP beamline at DLS.^12,21^ The same tensile rig was used as has been described in Section 2.4. The setup is illustrated in Figure 2d. Monochromatic beam energy was set to 85 keV with a spot size of 200 μm × 200 μm. At each loading step of tensile deformation, a large-area scan was conducted covering the central volume of the samples, shown in Figure 2b; five line scans contain 72 scanning points each. Debye–Scherrer patterns were recorded by a large 2D area diffraction detector Pilatus 2M CdTe (1475 × 1679 pixel, pixel size 172 μm × 172 μm). Based on the setup calibration from the Data Analysis WorkbeNch (DAWN) platform, the Rietveld analysis was performed using GSAS II.^23^ An example of the diffraction pattern and its refinement is shown in Figure 2e, showing the decent identification of both B19′ martensite and B2 austenite phases of the NiTi shape memory alloy. The texture of both phases was precisely quantified by the analysis routine in.^21^

## 3 Results and Discussion

### 3.1 Precipitation

**Figure 3:**
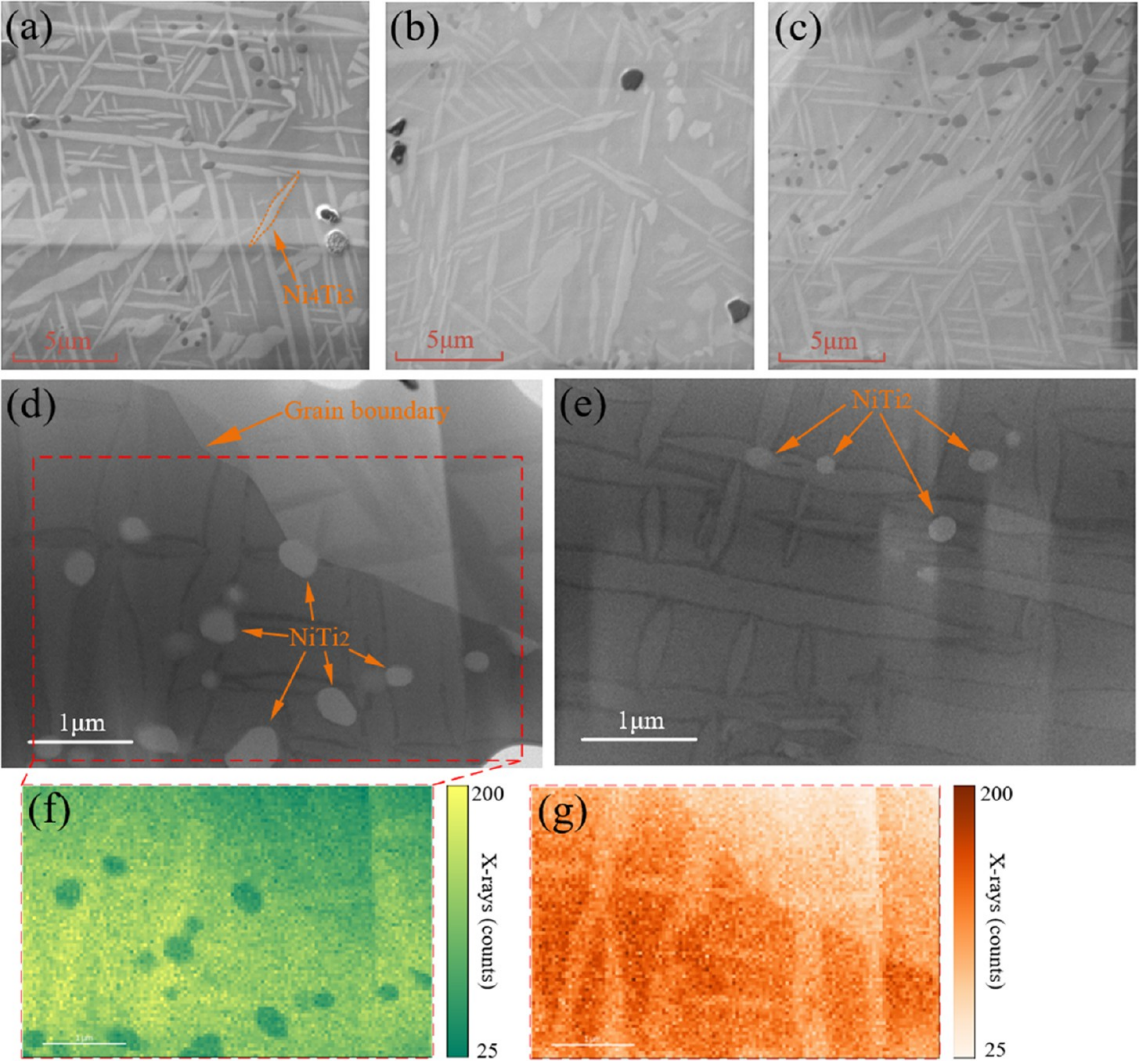
FIB-SEM imaging of precipitation in (a) AC, (b) HS, and (c) HR NiTi. TEM imaging of precipitation in (d) AC and (e) HS NiTi; the ⟨100⟩ lattice direction is perpendicular to the surface. TEM-EDX elemental mapping of Ni (f) and Ti (g) is shown by the dashed line area in (d). Signal intensity of Ni Ka at 7.48 keV and Ti Ka at 4.51 keV are shown here. Precipitation types are identified and tagged.

During heat treatment, remarkable precipitation activities take place in as-cast (AC), hot rotary swaged (HS), and hot-rolled (HR) NiTi materials. It can be observed from FIB-SEM imaging that precipitation is very similar among the three materials in terms of type, size, and orientation, as shown in Figure 3a–c for AC, HS, and HR, respectively. From TEM-EDX analysis of the Ni–Ti composition ratio in Figure 3d,e for AC and HS NiTi, respectively, it can be confirmed that lenticular-shaped structures are Ni_4_Ti_3_ precipitates, while the round-shaped ones are NiTi_2_ precipitates randomly embedded in the matrix and in the Ni_4_Ti_3_. Figure 3f,g demonstrates the X-ray intensity distribution in TEM-EDX elemental mapping of Ni and Ti, respectively. Due to the stress-free heat treatment condition, three orientations of Ni_4_Ti_3_ are homogeneously distributed, whose length ranges from 2 to 10 μm and aspect ratio around 10:1, which agrees well with previous study.^8^ From the observations, it can be concluded and assumed that precipitation is similar among the three NiTi materials in terms of the precipitate type, fraction, shape, size, and spatial distribution. Therefore, it is not considered a factor that causes the difference in the superelastic tensile loading behavior.

### 3.2 Superelastic Stress–Strain Response

**Figure 4:**
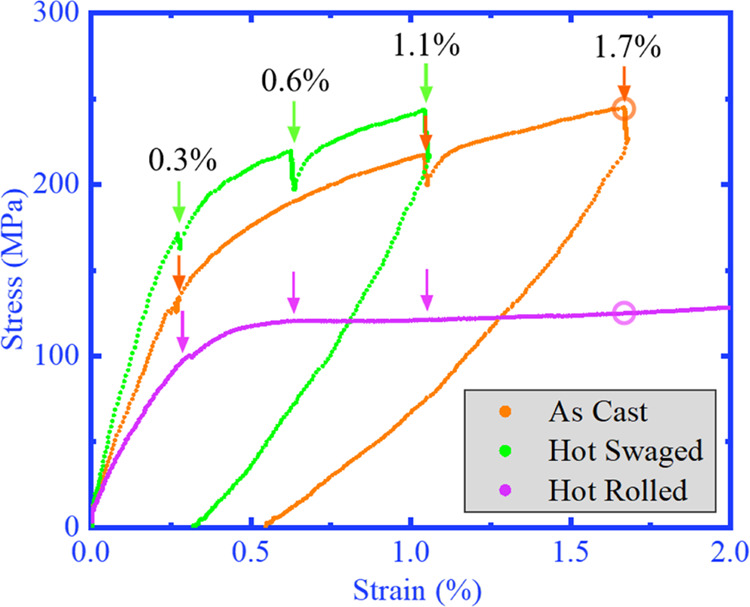
Superelastic stress–strain response during tensile loading. The strain levels at which the *in situ* μLaue scans were conducted are denoted by arrows, and the position of the *in situ* HE-XRD scans is denoted by hollow circles. The AC and HS curves were recorded during *in situ* μLaue experiment so that there is an obvious stress-relaxation at each scanning point, while the HR curve was recorded during *in situ* HE-XRD experiment without any visible stress drops thanks to the short acquisition time.

The superelastic tensile loading behaviors of the three materials are shown in Figure 4. Strain level is defined by Δ*L*/*L*, where Δ*L* refers to the extension measured by the extensometer and *L* is the original length of the sample. Although the strain level at which major phase transformation activity was triggered was almost the same value at around 0.3–0.4% for all three materials, the stress level shows a major difference: HS NiTi has the largest phase transformation stress of nearly 200 MPa, followed by AC NiTi at ∼150 MPa, and HR NiTi is merely ∼110 MPa. It is worth mentioning that cold-drawn NiTi wires with nanocrystallites possess a phase transformation stress level as high as 350 MPa.^8^ In addition, AC and HS NiTi exhibit incremental stress variation during superelasticity, while the HR NiTi shows a flat stress plateau. The cause of such diverse superelasticity is mainly due to the phase transformation kinetics, which is further discussed in Section 3.4.

### 3.3 Grain Structure: EBSD−μLaue Comparison

**Figure 5:**
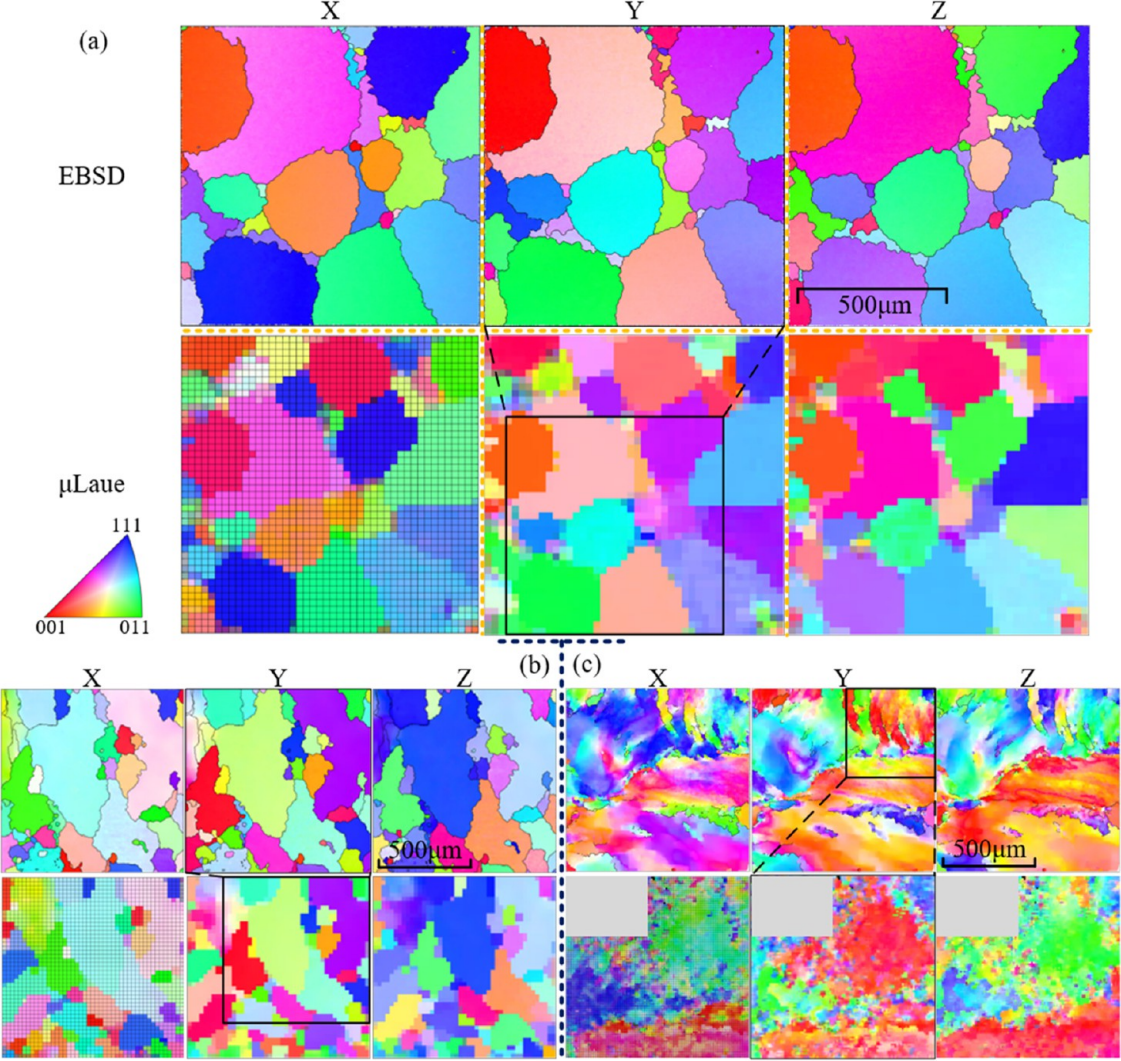
EBSD−μLaue lattice orientation map comparison of (a) AC, (b) HS, and (c) HR NiTi. The grid in the *X*-direction μLaue map indicates the positions of scanning points (pixel sizes): 44 × 44, 45 × 45, and 101 × 101 for AC, HS, and HR, respectively. Each grid is a scanning point. The step sizes are 30, 30, and 5 μm for AC, HS, and HR, respectively. The black box in the *Y*-direction μLaue map indicates the region of the EBSD map in (a) and (b), and vice versa in (c) as the μLaue map is smaller due to the complexity in the grain structure. The gray area in (c) is a nonindexed area due to the unknown detector error during the experiment.

The EBSD maps of AC, HS, and HR NiTi are of the same size of exact 1000 μm length square, as displayed in the upper row of Figure 5a–c, while the μLaue maps are displayed in the lower row. Inverse pole figures (IPFs) of three sample coordinate directions *X*, *Y*, and *Z*, as defined in Figure 2b, are arrayed from left to right.

Due to the visibility of the FIB marker in both SEM and optical microscopes, EBSD and Laue maps are conducted at precisely the same area, one of which overlaps the other, depending on the grain size observed from the EBSD map.

Both AC and HS NiTi have clearly identified grain structures: each grain is separated from its neighbors by high-angle grain boundaries (HAGBs). Despite the inhomogeneous distribution of grain size in both materials, the average grain size of AC NiTi is quantified as ∼400 μm, while that of HS NiTi is much smaller at ∼220 μm. The comparison of IPF maps between EBSD and μLaue indicates excellent orientation matches in all three directions, implying the correctness of the indexation of μLaue diffraction patterns, although disagreement may be observed at regions where small grains crowded together. These disagreements are mainly attributed to the difference of several orders of magnitude in penetration depth between EBSD and μLaue: the EBSD map is generated within a small interaction volume at the sample surface with a typical depth of 10–50 nm;^24^ by contrast, the energy of the synchrotron X-ray beam for μLaue allows a probing depth of 15–20 μm under the surface, very likely to illuminate another grain under the surface other than the one shown in the EBSD map.

The HR NiTi material possesses a smoothly changing grain structure with few HAGBs. Instead, low-angle grain boundary (LAGB) is more prevailing, which has been outlined by the thin black lines in the EBSD map. These thin lines rarely form a close loop. To some degree, the grain structure of HR NiTi can be regarded as a colossal grain, within which subgrain regions possessing different orientations are separated by LAGB. Because of this peculiarity of grain structure and the difference in probing depth, the μLaue map mismatches the EBSD map in fine details; however, the orientations (colors) obtained from the two techniques show a close match from a general point of view.

### 3.4 Charting the Phase Transformation Morphology

**Figure 6:**
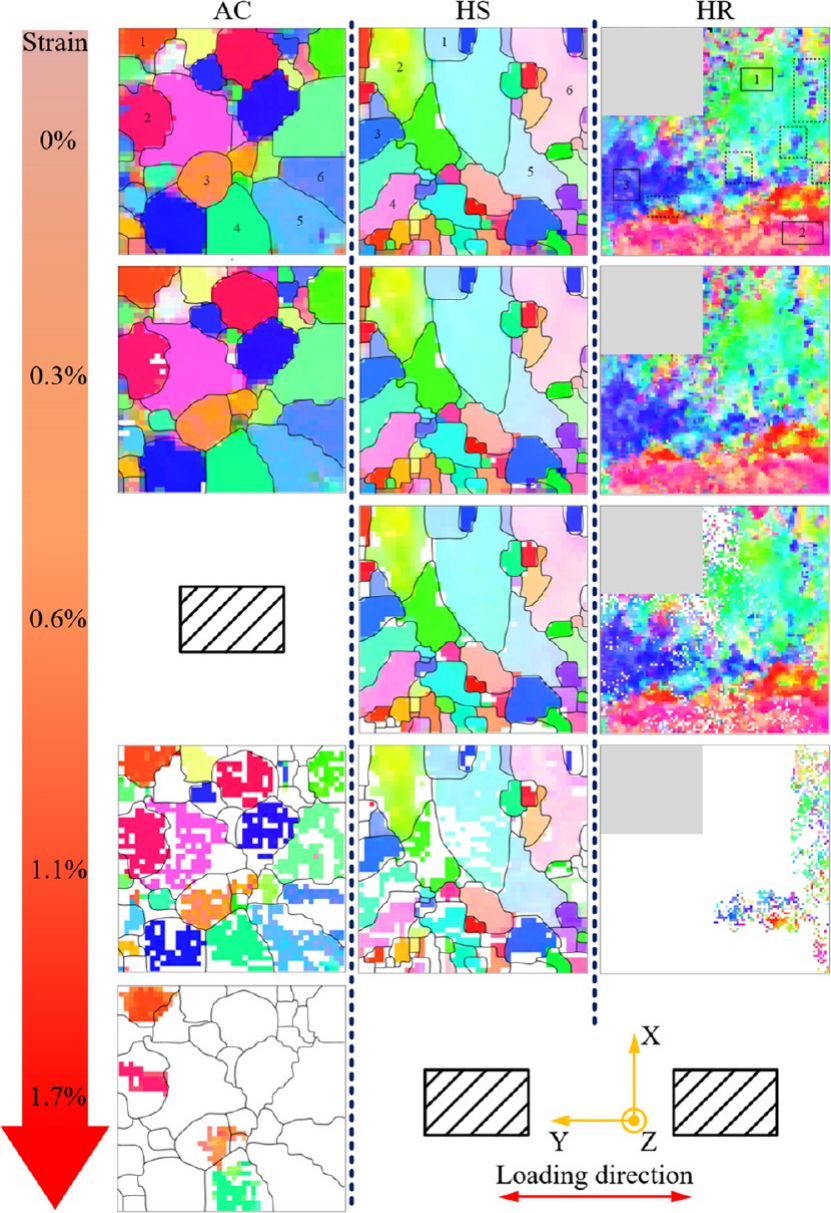
Evolution of μLaue orientation maps of AC, HS, and HR NiTi from the virgin state to the 1.7% strain. Three maps are not available, one at 0.6% and two at 1.7% strain. Sample coordinate is displayed at the bottom: loading direction is parallel to the *Y* axis. For demonstration of lattice rotation, six grains are numbered for AC and HS NiTi, and eight regions are marked by dashed/solid squares for HR NiTi at 0% strain.

The evolution of μLaue lattice orientation maps of AC, HS, and HR NiTi during superelastic loading is shown in Figure 6; the strain levels at which the maps were acquired are indicated by arrows in Figure 4.

Before tensile loading, the μLaue map of all three samples has nearly a 100% indexation rate for the B2 austenite phase. With the increment of strain levels, the indexation rate drops, leaving some blank area in the map, which is concluded to be closely linked to the formation of a large amount of stress-induced martensite phase.^25^ However, due to the intrinsic feature of the μLaue technique, the diffraction pattern is preferably originated from a single crystal illuminated by X-ray, having more than one crystal structure may cause misinterpretation in the indexation process. Considering the fact that multiple martensitic variants^26^ could emerge during stress-induced phase transformation, and their relatively small volume (i.e., needlelike structure^27^) compared to the size of the X-ray beam, these areas are left blank to avoid being indexed improperly.

During elastic loading from 0 to 0.3% strain, a slight elongation of the grain shape along the loading direction can be perceived on some colossal grains from AC NiTi (grain Nos. 3 and 5), although not obvious on HS NiTi with a smaller grain size. It can be easily observed from the strain level 0.3 to 1.1% that during superelastic loading, phase transformation happens preferentially at high-angle grain boundaries (HAGBs) in AC and HS NiTi, while for HR NiTi, which features low-angle grain boundaries (LAGBs), phase transformation happens randomly in certain regions but overall heterogeneous. This phenomenon is attributed to the concentration of elastic strain near HAGB, as will be discussed later in Section 3.5. At a 1.1% strain level, a large fraction of all three materials transforms to martensite. For AC and HS NiTi, phase transformation nucleates at HAGB and propagates to other areas, and this nucleation-propagation process continuously happens throughout the deformation. For HR NiTi, the triggering of nucleation is slow, as at 0.3% strain, the indexation rate is still 100%. However, its propagation is much quicker, so that a much larger proportion transforms to martensite at 1.1% strain. In all three materials, some grains/regions seem to be more rigid than others as they retain an untransformed state at a high strain level, e.g., grain Nos. 1–4 in AC NiTi.

The local color within individual grains keeps changing subtly throughout the loading process, indicating local lattice rotation, particularly on AC and HS NiTi, where local color variations become significant at higher strain levels in grains that originally have a relatively homogeneous color distribution. This phenomenon can be clearly observed from grain Nos. 1–6 in AC and HS NiTi. For instance, the No. 3 grain in AC NiTi shows a homogeneous orange color at 0% strain, upon loading small color variation becomes obvious among grids, and at 1.7% strain, the grid color ranges from dark orange to light orange. However, for HR NiTi, the local lattice rotation happens in a more complete manner, viz. the local color gradually becomes consistent with its neighboring regions, as demonstrated in the five regions marked by dashed squares. A quantitative analysis of individual grain lattice rotation is given in Section 3.6.

**Figure 7:**
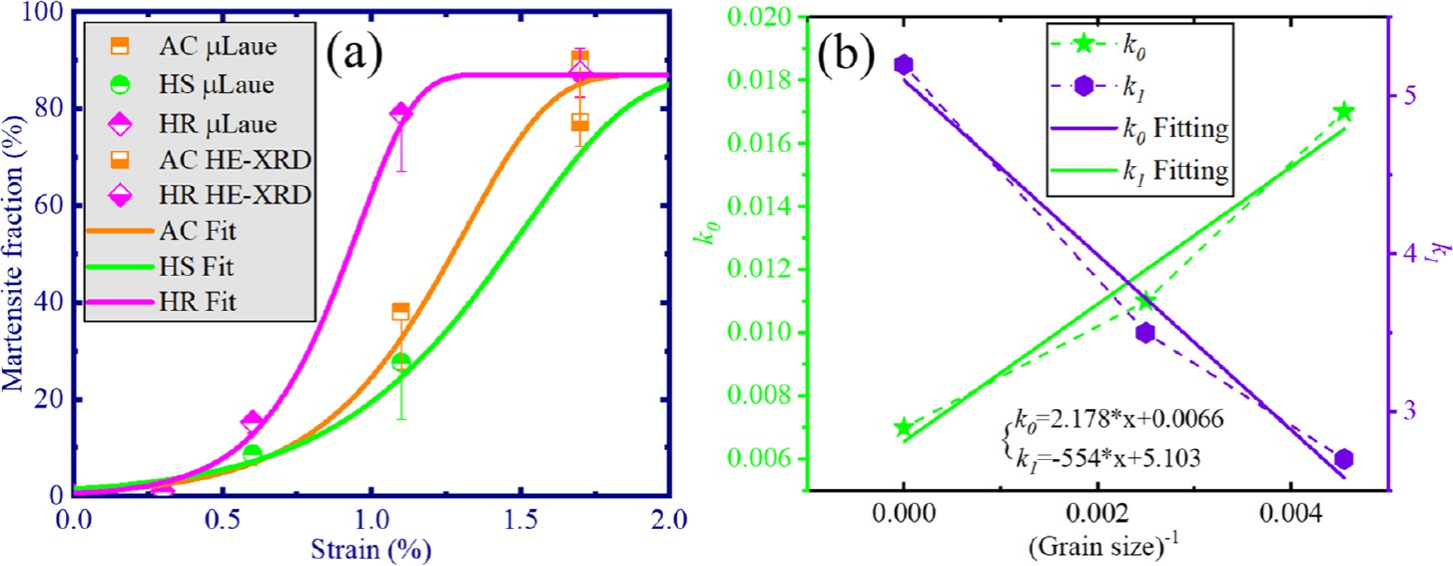
(a) Evolution of the martensite fraction during loading. The error bar for μLaue is estimated by comparison to the phase fraction quantified by the Rietveld method from the *in situ* HE-XRD experiment. (b) Nucleation and propagation kinetics as a function of the reciprocal average grain size.

The fraction of the martensite phase can be estimated from the ratio of the nonindexed area to the whole area at each strain level, as shown in Figure 7a. Since the martensite fraction obtained from the μLaue map can be overestimated because of the residual austenite,^28^ a complementary measurement is conducted via HE-XRD to estimate the error range. The nucleation and propagation kinetics of martensitic phase transformation can be quantified by a modified Johnson–Mehl–Avrami–Zifan equation proposed in^8^
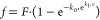
1where *F* is the saturated maximal fraction of martensite and *k*_0_ and *k*_1_ are parameters representing nucleation and propagation kinetics. The least-square fitted values are listed in Table 1 and plotted in Figure 7b as a function of the reciprocal of the average grain size. It is of great importance to note that the smaller the grain size, the higher the nucleation kinetics, and the lower the propagation kinetics. Nucleation kinetics determines the rate of nucleation of phase transformation in an aggregate of grains, while propagation kinetics determines the rate of propagation of phase transformation in an individual grain. This insight well explains why excellent superelastic performance is always achieved in nanocrystalline NiTi materials. Namely, these materials have a higher nucleation rate of new phases so as to accommodate the incremental strain level homogeneously throughout the whole piece (an aggregate of grains), rather than trying to propagate the new phase inside individual grains so that phase transformation takes place in a more inhomogeneous way. Because if phase transformation happens heterogeneously in the macroscale, those large austenite regions that are not transformed are more likely to be plasticly deformed. This conclusion is supported by the residual plastic strain of AC and HS NiTi after superelastic loading, as shown in Figure 4: AC NiTi exhibits a residual strain of ∼0.55%, while that of HS NiTi is ∼0.3%, which indicates that plastic deformation plays a bigger role in materials with larger grain sizes.

**Table 1:** Fitted Parameters *k*_0_ and *k*_1_: Nucleation and Propagation Kinetics

	mean grain size (μm)	*k*_0_	*k*_1_
HR NiTi	∞	0.007	5.2
AC NiTi	400	0.011	3.5
HS NiTi	220	0.017	2.7

On the other hand, higher propagation kinetics allows the material to accommodate the incremental strain level by pseudo-plastic phase transformation rather than by elastic deformation, thus resulting in a lower stress level. This matches well with the results in Figure 4, where HR NiTi exhibits the gentlest increase in stress level.

### 3.5 Spatial and Statistical Distribution of Elastic Strain

**Figure 8:**
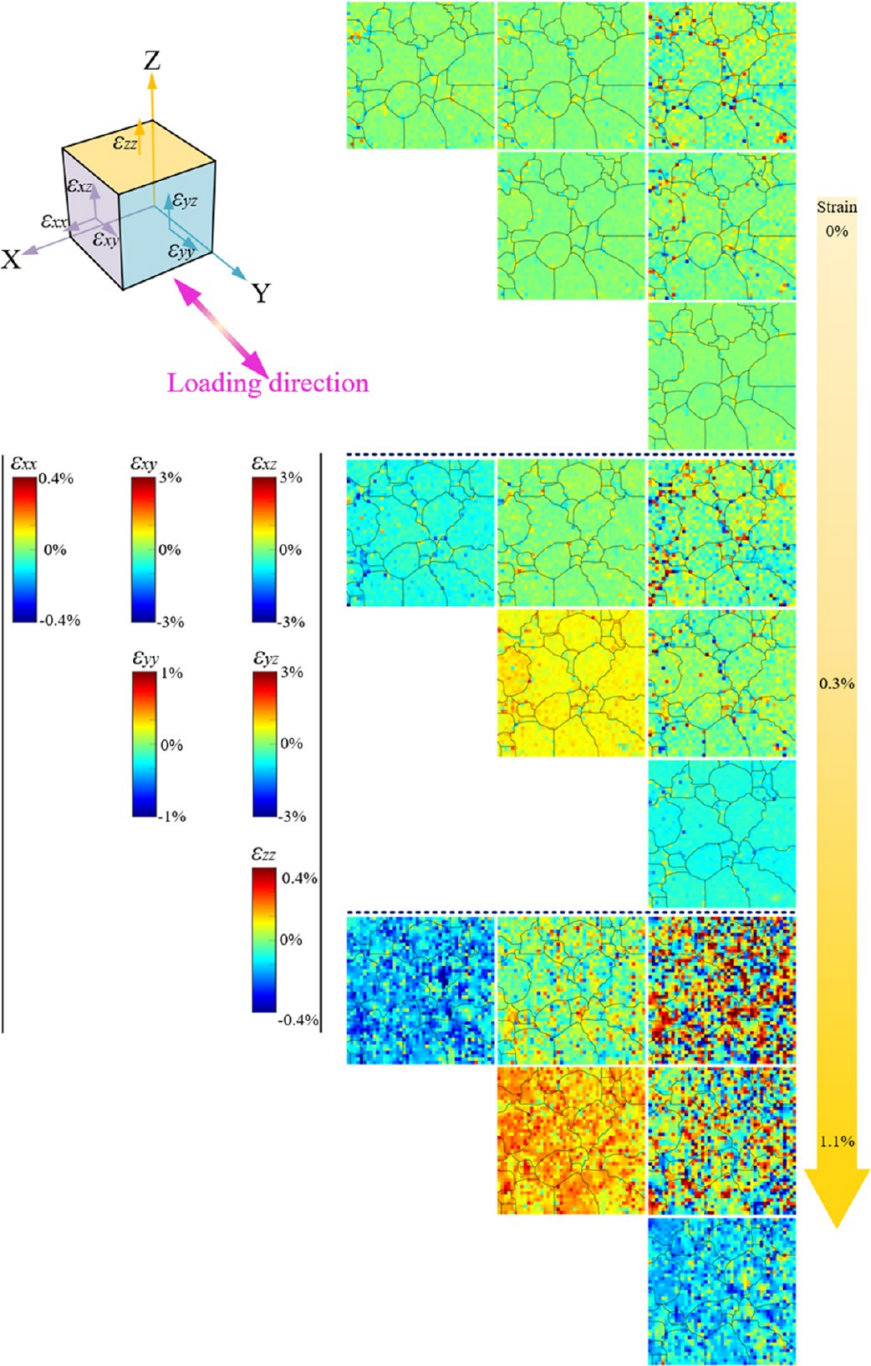
Evolution of the elastic deviatoric strain map of AC NiTi during superelastic tensile loading. The maps were measured at 0, 0.3, and 1.1% strain. The scale bars of components of the diagonal strain tensor are displayed on the left-hand side, with the component name sitting on top of the bar. The definition of each strain component is drawn on the top left infinitesimal cube, representing the local material volume illuminated by a micro X-ray beam. The grain boundaries are derived from μLaue lattice orientation maps in Figure 6. The coordinate system corresponds to the one in Figure 6. The loading direction is parallel to the *Y* axis. For a few scanning points where data is missing, the values are filled by linear interpolation.

**Figure 9:**
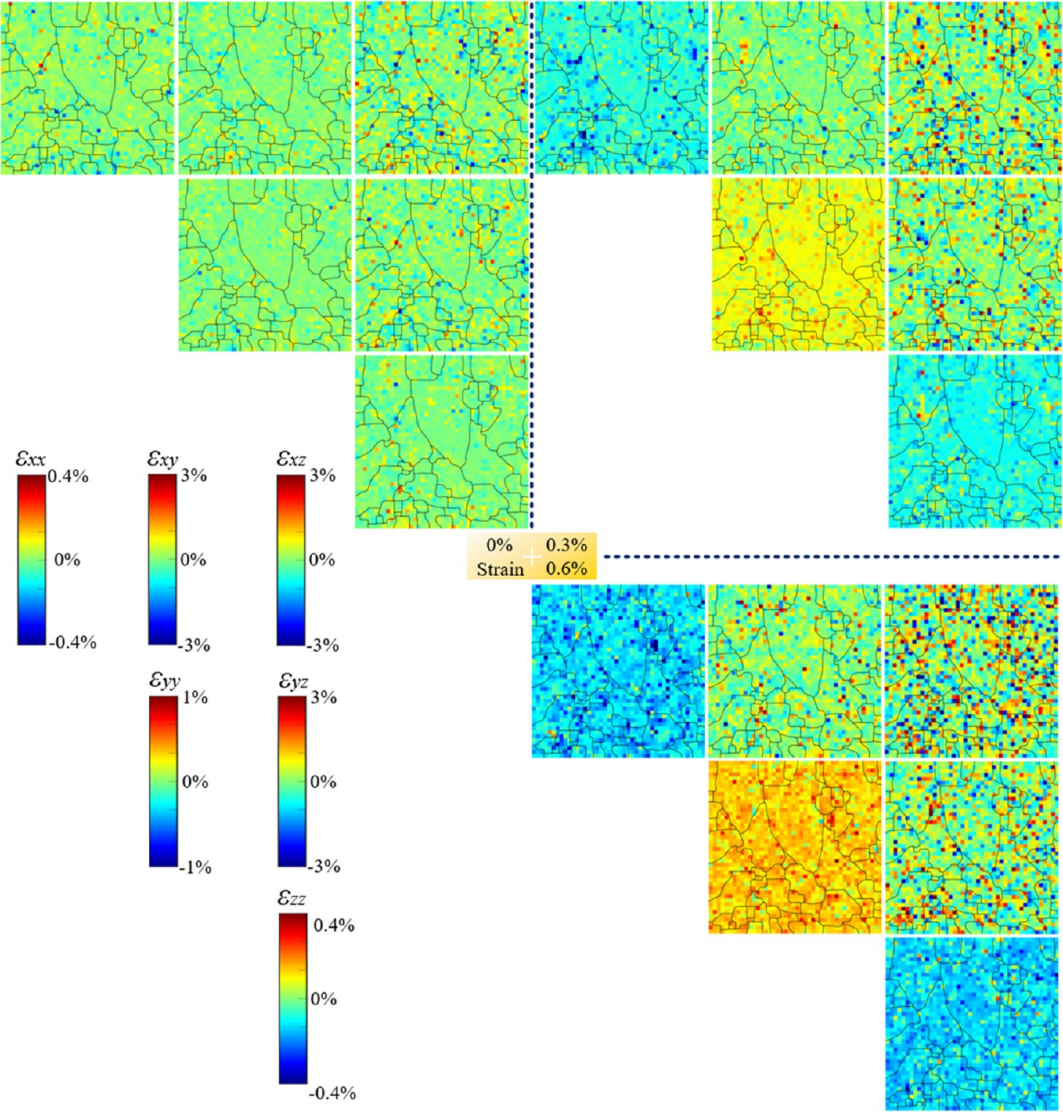
Evolution of the elastic deviatoric strain map of HS NiTi during superelastic tensile loading. The maps were measured at 0, 0.3, and 0.6% strain. For a detailed caption, please refer to that of Figure 8.

**Figure 10:**
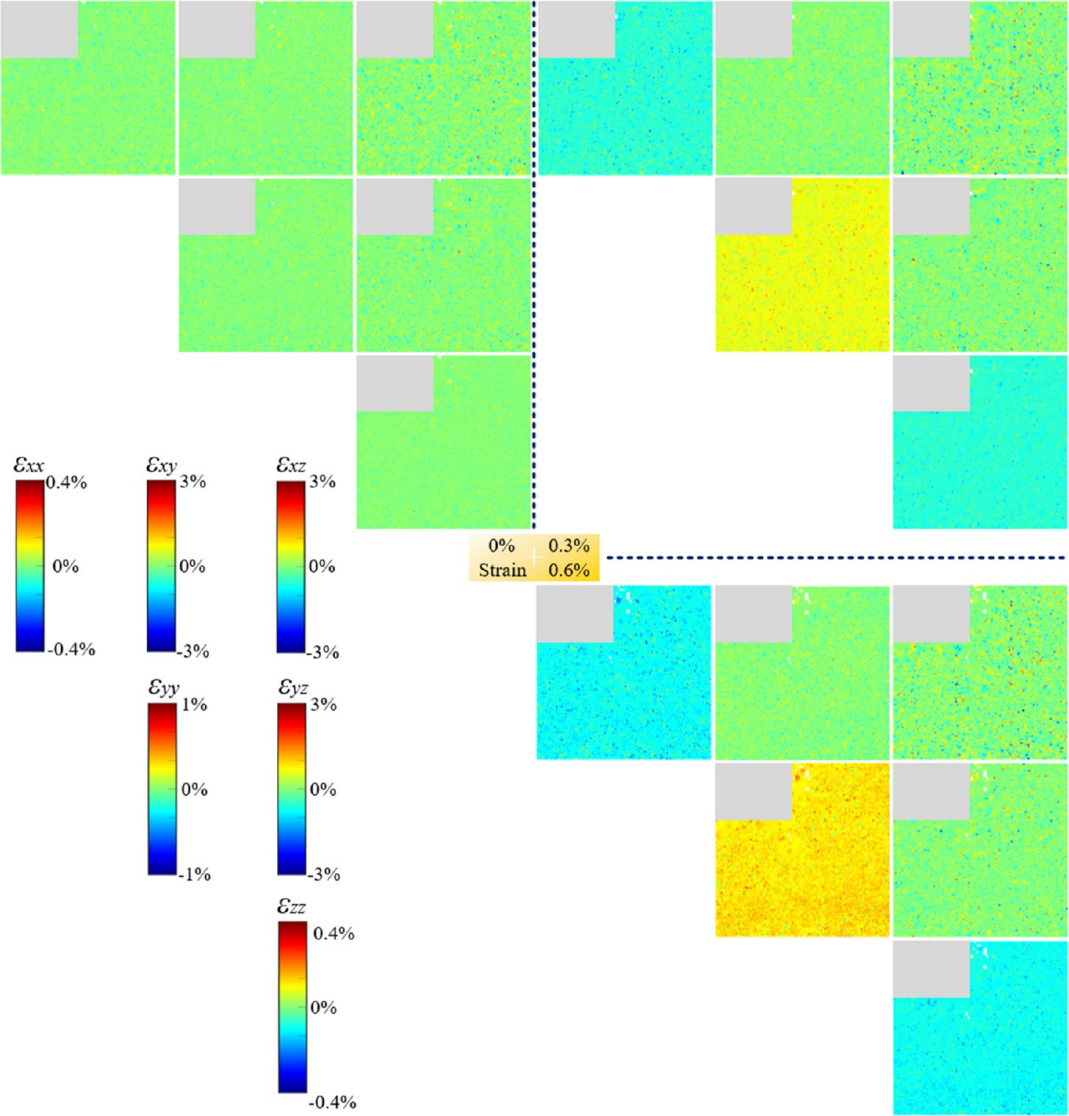
Evolution of the elastic deviatoric strain map of HR NiTi during superelastic tensile loading. The maps were measured at 0, 0.3, and 0.6% strain. The gray region is a nonindexed area due to unknown detector error during the experiment. For a detailed caption, please refer to that of Figure 8.

The elastic deviatoric strain can be precisely quantified from μLaue diffraction patterns.^11^ Each pattern gives all six components of the diagonal strain tensor at that scanning point. Therefore, the strain map can be established for each of the six strain components, as shown in Figures 8–10 for AC, HS, and HR NiTi, respectively. Note that the loading direction is parallel to the direction of ε_*yy*_. It should also be pointed out that elastic strain is equivalent to stress, so the two concepts are considered the same in the text below.

In the case of AC NiTi, the material exhibits small residual stress at the initial state, although some stress concentration can be seen particularly at HAGB. The majority of strain values lie within the range ±0.05%. However, some variations can be observed from shear strain components ε_*xz*_ and ε_*yz*_, whose direction is perpendicular to the surface. Upon loading to 0.3% strain, which is at the end of the elastic region in the macroscopic stress–strain curve, the overall color of the ε_*yy*_ map changes to yellow (∼0.25% elastic strain), indicating tensile force in the *Y*-direction, and those of ε_*xx*_ and ε_*zz*_ maps change to light blue (<0.1% strain), indicating compression. Stress concentration intensifies in all of the six strain maps. Interestingly, most concentrations still happen near HAGB, which explains the phenomenon in Figure 6 that phase transformation preferentially nucleates at HAGB. Namely, phase transformation is more easily triggered at high stress regions near HAGB. At a macroscopic strain level of 1.1%, local strain variation significantly broadens, particularly the shear strains, which show a range of nearly ±2.5%. However, the exact strain value may be unreliable at this state due to the heavy distortion of reflections in the μLaue diffraction pattern, which leads to error in refinement. Another interesting phenomenon that is worth mentioning is the difference in stiffness among grains during the loading process. For instance, in the ε_*yy*_ map, in grain Nos. 2–4 (Figure 6), they exhibit a deeper yellow color (especially grain No. 2) than other grains at 0.3 and 1.1% strain. This means these grains are stiffer and undertake higher stress. They are also the three among the only four grains that survive at 1.7% strain. On the contrary, grain No. 5 exhibits a lighter yellow color at 1.1% strain, representing lower stiffness.

In the case of HS NiTi, the general observations agree well with those from AC NiTi, where the stress concentration occurs mostly at HAGB, and the stress concentration intensifies throughout the loading process. Nevertheless, via comparison between AC and HS NiTi, some extra findings can be observed. First, in the virgin state, HS NiTi shows more residual stress than AC NiTi, which is attributed to the metal forming process, although careful heat treatment has been conducted. Second, at 0.3% strain, HS NiTi exhibits a denser stress concentration due to the higher amount of HAGBs in HS NiTi than that of AC NiTi. This explains the higher phase transformation nucleation kinetics in the material with denser HAGB in Section 3.4.

HAGB between two neighboring grains indicates a large misorientation of crystallographic direction, usually greater than ∼15°. Because of this incoherency in the lattice structure, the vast majority of stress concentration occurs at HAGB both in the initial state and during superelastic loading.

The HR NiTi material shows a completely different spatial distribution of strain. In the initial state, the residual strain is exceptionally low, ranging from ±0.025% for the majority of the ε_*yy*_ map. Because the grain structure is dominated by LAGB, there is no obvious stress concentration upon loading to 0.3% strain and further to 0.6% strain. For ε_*yy*_, some individual points show a significantly higher stress than the others, but they are not clustered together as those in AC and HS NiTi, so strain distribution seems to be rather homogeneous over the entire map. This phenomenon explains the higher phase transformation propagation kinetics in an LAGB-dominated NiTi.

**Figure 11:**
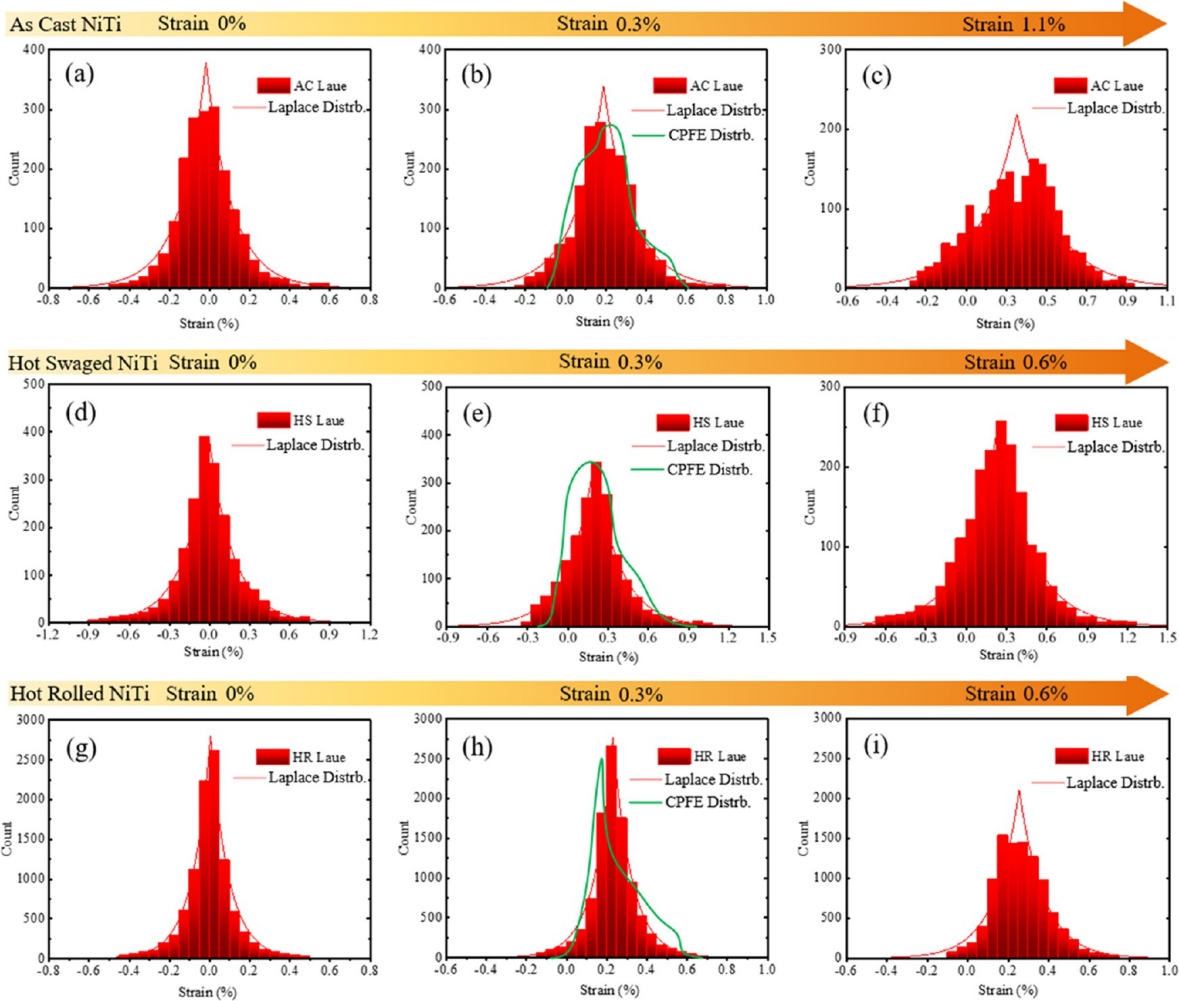
Evolution of statistical distribution of the elastic strain ε_*yy*_ for AC (a–c), HS (d–f), and HR (g–i) NiTi during superelastic tensile loading. The red curve is the fitted Laplace distribution. The green curve is the profile shape obtained from CPFE simulation in Appendix B.

The statistical distribution of elastic strain ε_*yy*_ has been extracted from the maps in the previous figures and are plotted in Figure 11a–i for all three materials.

In the virgin state, HS NiTi possesses the highest residual strain, as seen from the wide peak range, followed by AC NiTi, while the strain of HR NiTi mostly concentrates within ±0.025%. Despite that, it is found that the statistical strain distribution of all three materials matches well with the Laplace distribution, as outlined by the red line. Upon loading to 0.3% strain, the distribution profile becomes asymmetric for AC and HS NiTi, where the left side of the peak becomes steeper and the right side extends to a much higher strain level than the average. However, the profile of HR NiTi still remains in Laplace distribution, and there is no significant broadening of the peak width, except that the peak center shifted to ∼0.24% strain. A CPFE simulation was conducted to verify the distribution profile shape at this strain state. For detailed simulation settings, please refer to Appendix B. It can be seen that the asymmetry feature of the strain distribution is well captured by the model, in which the distribution profile of HS NiTi exhibits a more gradual slope extending to a higher strain level. At 0.6% strain, the strain distribution of HR NiTi changes to an asymmetric profile. At 1.1% strain, AC NiTi exhibits a bimodal distribution.

When the three materials are loaded to the same strain level, HS NiTi always shows the highest maximal elastic strain. For example, at 0.3% strain, HS NiTi shows the maximal elastic strain of ∼1%. Similar observations can be made at 0.6% strain between HS and HR NiTi. The strain distribution of HR NiTi is always the narrowest of all. This diversity in strain distribution is closely linked with the grain structure, where HAGB contributes to the broadening of the distribution range and the increase in the maximal strain value, while LAGB has less such effect. Subsequently, such a difference in strain distribution causes the different phase transformation behaviors among the three materials, as discussed in Section 3.4, that a higher maximal strain and a wider strain distribution range results in the higher nucleation kinetics and lower propagation kinetics, and, contrariwise, it is also true.

It should be noted that the mean elastic strain is not necessarily equal to the total strain due to phase transformation happening at the early stage of superelastic loading. At a total strain of 0.3%, HR NiTi has the largest proportion of the new phase, and hence the lowest value of the mean elastic strain (∼0.22%), because the rest of the total strain is accommodated by the pseudo-plastic phase transformation process. Similarly, HS NiTi exhibits the highest value of elastic strain (∼0.25%).

### 3.6 Lattice Rotation of Individual Grains

**Figure 12:**
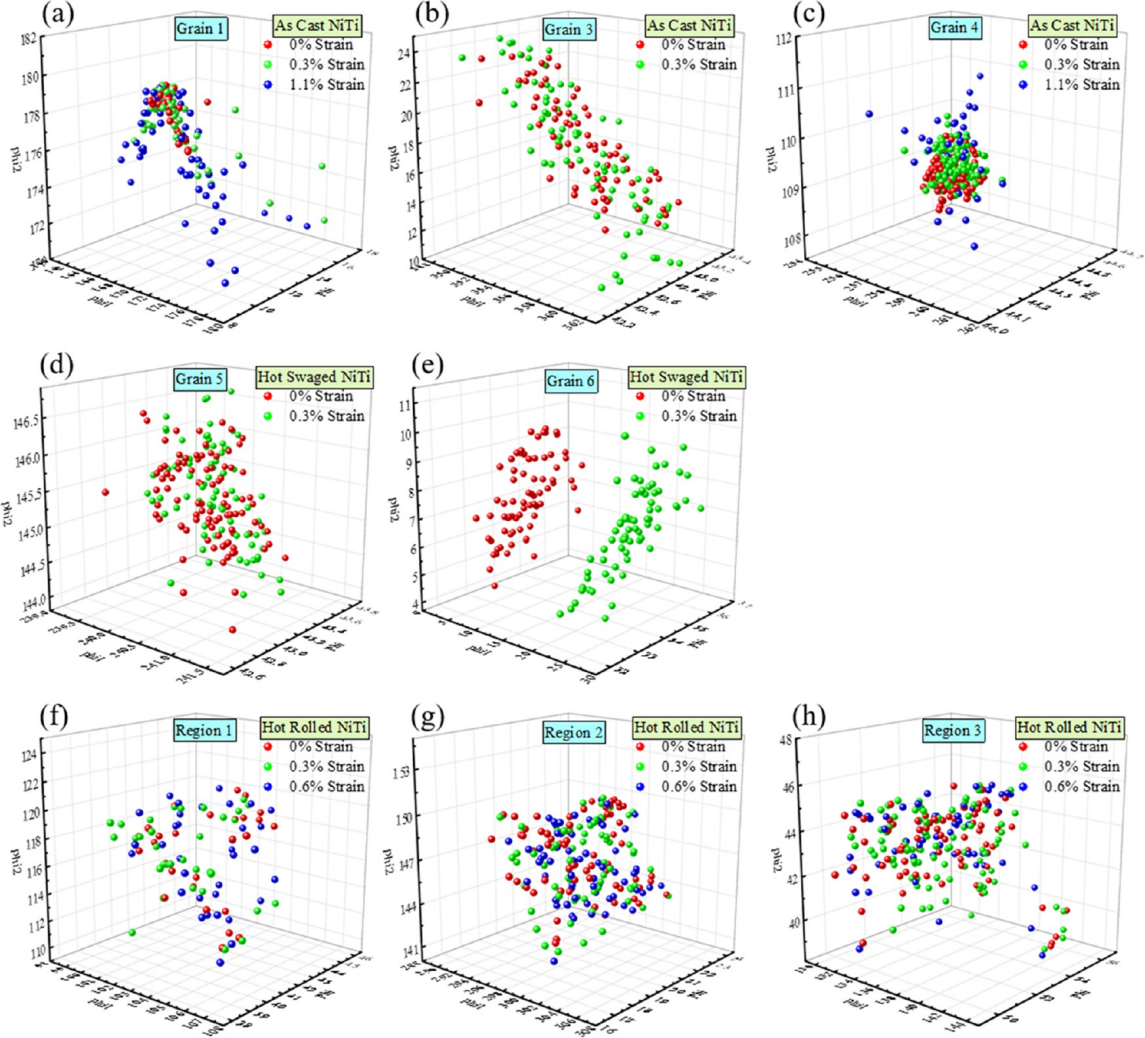
Lattice rotation of selected grains from the three materials: AC (a–c), HS (d, e), and HR (f–h) NiTi. Each sphere represents one scanning point. Different colors represent different loading steps. Three axes represent three Euler angles in Bunge notation (ϕ_1_,Φ,ϕ_2_).

Certain grains/regions are selected from the three materials and plotted in Figure 12a–h. These grains and regions are labeled in Figure 6. Each colored sphere represents a μLaue scanning point.

For AC NiTi, three of the four stiffest grains are chosen, namely, grain Nos. 1–4. Interestingly, though these three grains survived at a high strain level of 1.7%, they exhibit diverse rotation modes. Grain No. 1 starts with a cluster of orientations in the middle; upon deformation, the lattice splits into two parts, rotating to two different directions, as can be seen from the evolution from red points to green points and finally to blue points. On the contrary, grain No. 4 rotates to three different directions and grain No. 3 rotates to one direction. Despite the various numbers of rotation directions, one thing is common: some parts of the grain remain in their original orientation. Therefore, this rotation mode can be categorized as multiextension rotation.

For HS NiTi, the two selected grains behave differently. No obvious rotation direction can be observed in grain No. 5 since lattice orientations seem to be constrained within a certain volume upon deformation, though slight rotations may happen locally. In comparison, grain No. 6 shows a complete rotation where the entire cluster of orientations shift to another region, as can be seen in Figure 12e. Since the whole grain is rotated in a homogeneous manner, this rotation mode can be categorized as rigid rotation.

For HR NiTi, the three regions show similar rotation behaviors as shown in Figure 12d. Upon deformation, the orientations change slightly from place to place but are always confined within the same limitation. Since all three regions exhibit a similar behavior, it can be assumed that this rotation mode is dominant in the LAGB structure in the NiTi alloy, which can be termed nondispersive rotation.

### 3.7 Texture Evolution and Phase Transformation Preference

**Figure 13:**
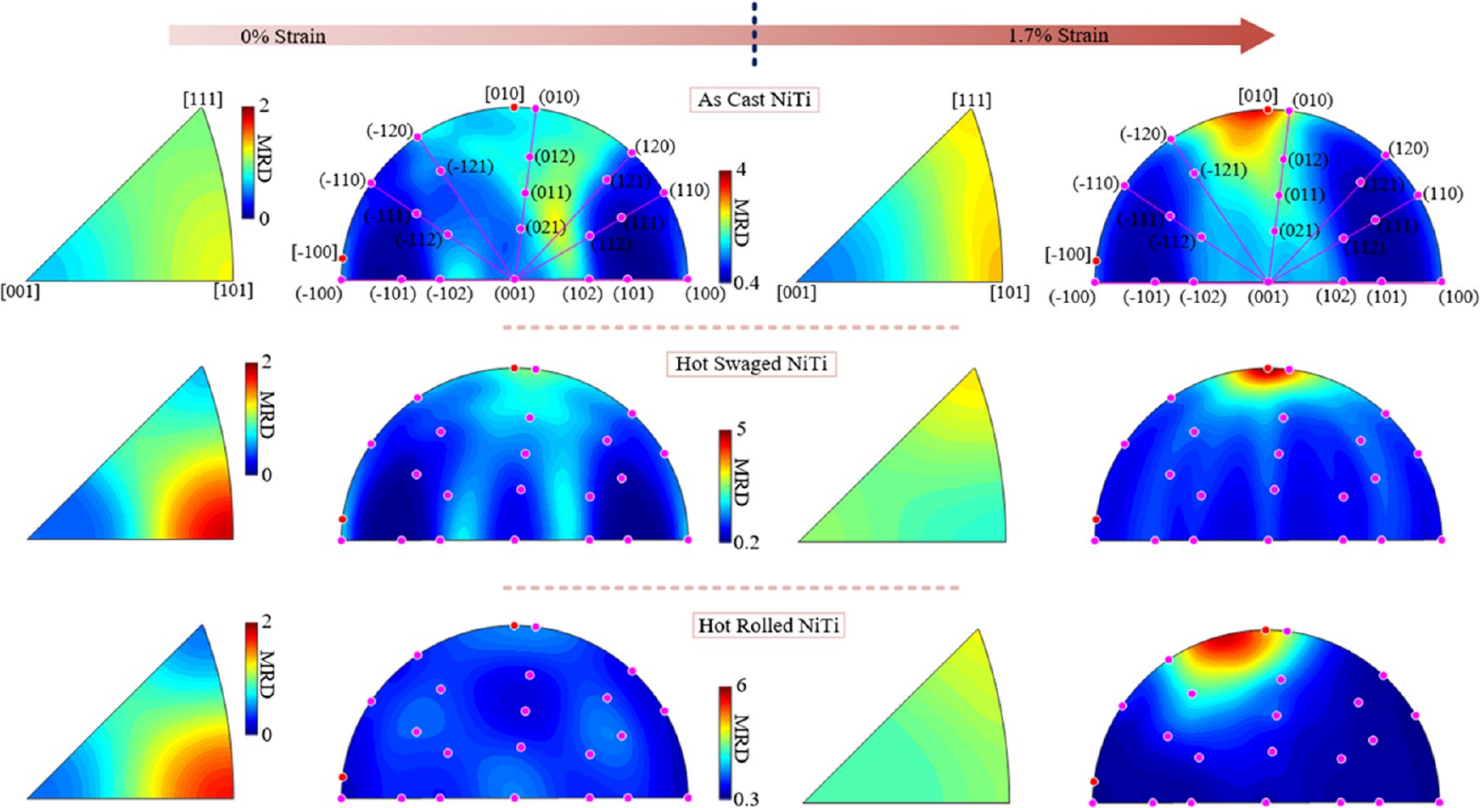
Texture evolution of AC, HS, and HR NiTi during deformation. The inverse pole figure is plotted in the loading direction (*Y* axis in Figure 2). The same scale bar is used for one phase, but varies in different materials.

The mesoscale texture information is obtained from the *in situ* HE-XRD experiment for all three materials. Data was recorded in the virgin state and 1.7% strain. The refined texture is shown in Figure 13. The inverse pole figure is plotted in the *Y*-direction in the coordinate system defined in Figure 2. The *Y*-direction is parallel to the loading direction. Although the tensile loading was conducted above the *A*_f_ temperature, some residual martensite is present in the material; thus, the texture of both phases can be refined at 0% strain.

In the initial state, AC NiTi possesses a weak B2 phase texture of ∼1.2 MRD, while HS NiTi and HR NiTi show a stronger [101] texture. After loading to 1.7% strain, the strong texture disappears in all three materials, and the texture reorientates to [111], but not as strong as in the original state. Generally, for all three materials, the texture of B2 austenite in the loading direction is weakened upon loading, exhibiting a more homogeneous distribution.

Regarding martensite, the initial texture of the three materials was different. However, the texture evolution shows a similar tendency upon deformation, where the texture concentrates in the [010] direction at a high strain. This phenomenon agrees well with recent studies on a nanocrystalline NiTi wire.^1,4^ This behavior of the martensite phase may be related to the naturally preferred detwinning mode to accommodate the applied strain.

Overall, combined with the observations on local lattice rotation in Section 3.6, it can be concluded that the texture evolution of the B2 austenite phase is not strongly dependent on the lattice rotation mode and the grain structure. Besides that, the texture evolution of B19′ martensite is also not strongly dependent on the grain structure. All three NiTi materials show a similar texture evolution mode in both phases.

## 4 Summary and Conclusions

In the present study, NiTi shape memory alloys of different grain structures have been produced using novel forming techniques. The grain structure in as-cast (AC) NiTi and hot swaged (HS) NiTi is composed of high-angle grain boundaries (HAGBs) of different grain sizes: ∼400 μm for AC NiTi and ∼220 μm for HS NiTi. Meanwhile, the grain structure in hot-rolled (HR) NiTi is dominated by low-angle grain boundaries (LAGBs), and very few HAGBs can be observed. These three materials exhibit diverse superelastic behaviors, and the insights into such discrepancy is given via conducting *in situ* synchrotron X-ray μLaue diffraction and powder diffraction experiment. This study serves as guidance and opens up possibilities to customize the superelasticity of NiTi shape memory alloys by means of grain structure engineering.

Conclusions are summarized as below:

The precipitation is similar among the three materials in terms of precipitate type, size, and orientation distribution. The majority is the Ni_4_Ti_3_ precipitates, and the NiTi_2_ precipitates are randomly embedded in the matrix and in the Ni_4_Ti_3_.

Upon deformation, slight elongation of the grain morphology can be observed in HAGB NiTi. Also, phase transformation happens preferentially near HAGBs, while for the LAGB structure, phase transformation happens randomly but overall heterogeneous.

The stress-induced phase transformation kinetics is quantified by a proposed equation consisting of two kinetics parameters. It is found that the smaller the grain size, the higher the nucleation kinetics, and the lower the propagation kinetics.

Upon loading, most stress concentration happens near HAGBs for all six components of the elastic deviatoric strain tensor. In the HAGB structure, the stress state varies among different grains. Nevertheless, no obvious stress concentration can be observed in the LAGB structure during loading. The stress concentration phenomenon well explains the physical significance of the two kinetics parameters.

In the virgin state, the statistical strain distribution of all three materials matches well with Laplace distribution, while it may become asymmetric during loading. Upon deformation, HAGB contributes to the broadening of the distribution range and the increase in the maximal strain value, while LAGB has less such effect.

Three grain lattice rotation modes are identified and termed as multiextension rotation, rigid rotation, and nondispersive rotation. The rotation mode in the HAGB structure is a mixture of the three, while in LAGB, the dominant mode is nondispersive rotation.

The texture evolution of both B2 austenite and B19′ martensite is not strongly dependent on the grain structure.

## Appendix A. Grain Structure Engineering Methods

The manufacturing routes for grain structure engineering are depicted in Figure S1.

Hot rotary swaging (HS) is a near-net plastic deformation method used to process solid and hollow workpieces of variable cross sections using high-speed action of a set of dies. As a relatively rapid and low-cost industrially applied continuous manufacturing process, HS features advantageous stress–strain conditions that enable one to impose very high shear strain into the processed material. HS can favorably be used to manufacture metals with ultrafine grain structures. It can be applied to manufacture challenging materials, such as metals with low plasticity (e.g., W, Mg), powder-based pseudo-materials, and composites. Since the process imparts gradient stress/strain distribution across cross sections of processed workpieces, it also provides a solution for the preparation of gradient microstructures^1^ and residual stress distribution.^29−33^ The NiTi workpiece was gradually swaged down from the initial diameter of 20 mm to the final diameter of 12.5 mm. An induction heating system was applied during swaging. Corresponding diagrams of deformation forces detected during the individual swaging passes are shown in Figure S2. It is evident that the slopes of increase in the swaging force before and after the swaging time of ∼2 s were steep for all three passes. The total reduction ratio upon HS was 0.94.

Hot rolling was executed using a two-high rolling mill stand with the diameter of rolls of 180 mm. To reduce thermal losses during rolling, a higher rotational speed of the rolls was used (40 RPM). Rolling was performed in a total of eight passes with identical rolling reductions in each pass. The deformation force during the individual passes gradually increased due to the incremental imposed strain, as can be seen from Figure S3. The rolling force developments during the individual passes exhibited a nonuniform behavior. The maximal measured deformation force for the first pass was 43 kN, while for the second pass the value increased up to 50 kN. A similar increasing trend was monitored for the third pass. Nevertheless, after this pass, the increasing trend changed. The fourth rolling pass featured an absolute maximal force of ∼72 kN, and subsequent passes exhibited an opposite trend, i.e., the next three passes featured gradual decreases in the maximum achieved rolling force. The decreasing trend in the maximal force values was caused by the development of dynamic restoration processes, which plays an important role during these rolling passes. For the last pass, an increase in the rolling force was observed, which can be attributed to work-hardening dominating over structure-softening. The total logarithmic strain of the workpiece was 0.625.

## Appendix B. CPFE Modeling

Crystal plasticity finite element (CPFE) simulation was conducted using a strain-controlled model in our recent study.^34^ The homemade Fortran code was imported in Abaqus 2021 via the UMAT portal.

To prepare the crystal model properties, EBSD maps of the three materials were imported into DREAM.3D software.^35^ Those EBSD maps display the grain structure at the surface layer, while that underneath is unknown. Because the thickness of the flat dog-bone-shaped sample is 1 mm, which is exactly the same dimension as the width and height of the EBSD maps, a columnar grain structure is assumed, and the crystal model is a 1 mm × 1 mm × 1 mm cube, as illustrated in the upper row in Figure S4. The crystal models are colored in the IPF *Y*-direction, identical to the EBSD maps in Figure 5.

The uniaxial tensile loading is applied in the X-direction from 0% strain to 0.3% strain. The loading process was set to completely fall within the elastic region without any plastic activity. This is confirmed by checking the model output that the plastic strain at all integration points is equal to zero. The C3D8 element configuration was used, and the meshing strategy is shown in the lower row in Figure S4. Periodic boundary condition was set using an Abaqus plugin tool EasyPBC. The elastic constants for the B2 austenite phase were determined from the literature,^36^ viz *C*_11_ = 169, *C*_12_ = 138, and *C*_44_ = 40.

The simulation result is displayed in Figure S4. The statistical distribution of ε_*xx*_ was exported from all integration points and is plotted in the histogram. The profile of the histogram was fitted by a kernel smooth function, which is also known as the kernel density estimation (KDE), to capture the precise shape profile. The shape profile is corrected by the following function: ε′ = *A*·(ε – ε_mean_), where ε_mean_ is the mean value of the entire strain distribution; the fitting parameter *A* is determined to be 3.5, 4.4, and 4.1 for AC, HS, and HR NiTi, respectively.

## Supporting Information Available

Grain structure engineering routes and equipment (Figure S1); swaging forces measured during NiTi HS (first, second, and third passes) (Figure S2); rolling force development during hot rolling (Figure S3); crystal models and simulation results of the three materials; the coordinate system is given at the bottom left corner (Figure S4) (PDF)

## Notes

The raw/processed data required reproducing these findings cannot be shared at this time as the data also forms part of an ongoing degree study.
