# Supplementary material for: Grain Structure Engineering of NiTi Shape Memory Alloys by Intensive Plastic Deformation
Source: ACS Appl Mater Interfaces. 2022 Jun 27;14(27):31396–410. doi: 10.1021/acsami.2c05939 (PMC9284517; doi:10.1021/acsami.2c05939)
Supplement: Supplementary file 1 — am2c05939_si_001.pdf [file am2c05939_si_001.pdf]

Supporting Information

Grain Structure Engineering of NiTi Shape Memory Alloys by Intensive Plastic Deformation

Zifan Wang[a,*], Jingwei Chen[a], Radim Kocich[b], Samuel Tardif[c,d], Igor P. Dolbnya[e], Lenka Kunčická[b], Jean-Sébastien Micha[c,d], Konstantinos Liogas[a], Oxana V. Magdysyuk[e], Ivo Szurman[f], Alexander M. Korsunsky[a,*]

[a] *MBLEM, Department of Engineering Science, University of Oxford, Oxford OX1 3PJ, U.K.*

[b] *Faculty of Mechanical Engineering, Brno University of Technology, Technická 2896/2, Brno 61669, Czech Republic*

[c] *Université Grenoble Alpes, CEA-Grenoble/IRIG, 38043 Grenoble Cedex 9, France*

[d] *CRG-IF BM32 Beamline, European Synchrotron Radiation Facility, 38043 Grenoble Cedex 9, France*

[e] *Diamond Light Source, Harwell Campus, Oxfordshire, OX11 0DE, U.K.*

[f] *Faculty of Materials Science and Technology, VŠB-Technical University of Ostrava, Ostrava 8, Czech Republic*

*Corresponding author. E-mail:

zifan.wang@exeter.ox.uk

alexander.korsunsky@eng.ox.ac.uk

Supplementary Figures

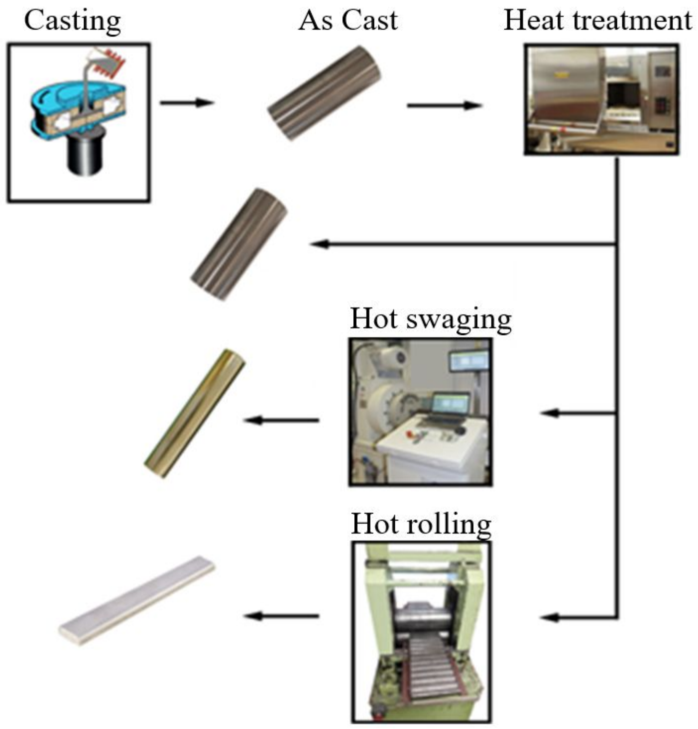

Figure S1. Grain structure engineering routes and equipment.

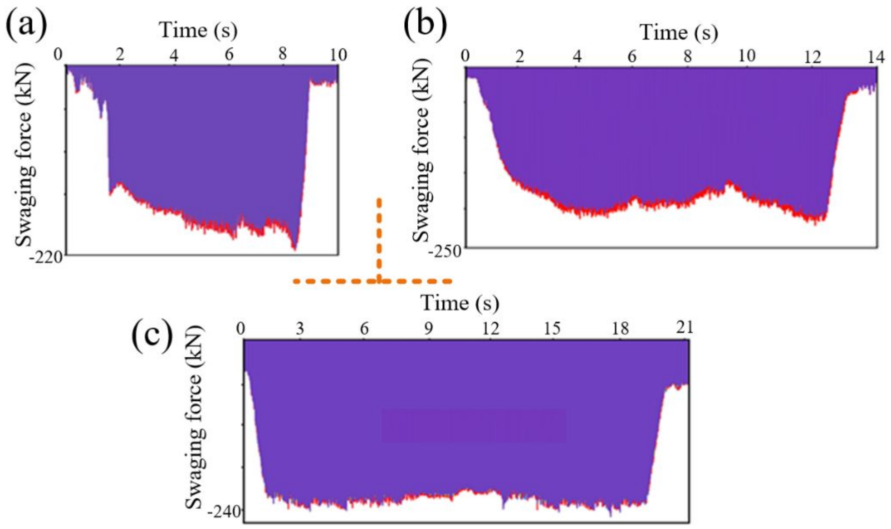

Figure S2. Swaging forces measured during NiTi HS. (a) First pass. (b) Second pass. (c) Third pass.

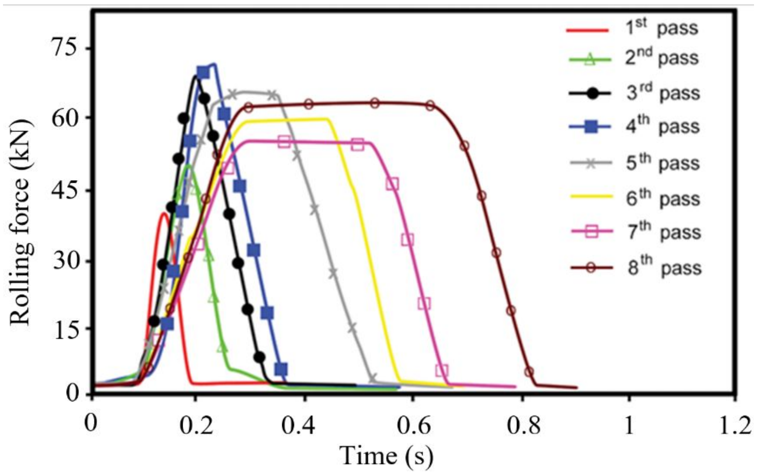

Figure S3. Rolling force development during hot rolling

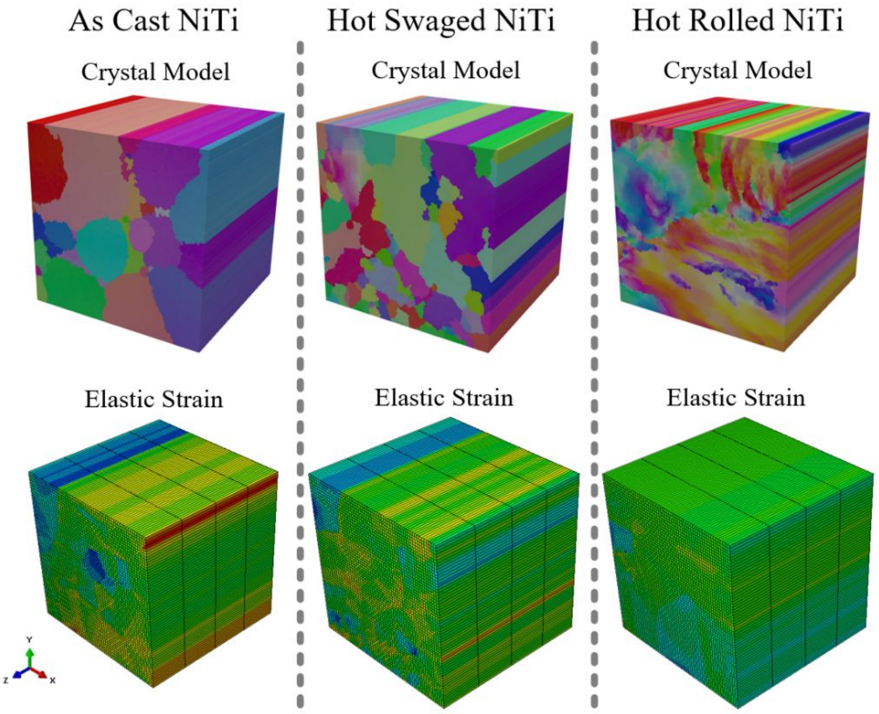

Figure S4. Crystal models (upper row) and simulation results (lower row) of the three materials. Coordinate system is given at the bottom left corner.
